# A Robust Real Time Direction-of-Arrival Estimation Method for Sequential Movement Events of Vehicles

**DOI:** 10.3390/s18040992

**Published:** 2018-03-27

**Authors:** Huawei Liu, Baoqing Li, Xiaobing Yuan, Qianwei Zhou, Jingchang Huang

**Affiliations:** 1 Science and Technology on Microsystem Laboratory, Shanghai Institute of Microsystem and Information Technology, Chinese Academy of Sciences, Shanghai 201800, China; liuhuawei@mail.sim.ac.cn (H.L.); libq@mail.sim.ac.cn (B.L.); 2 University of Chinese Academy of Science, Beijing 100049, China; 3 College of Computer Science and Technology, Zhejiang University of Technology, Hangzhou 310023, China; zqw@zjut.edu.cn; 4 IBM-Research China, Shanghai 201203, China; hjingc@cn.ibm.com

**Keywords:** robust, DOA estimation, MEMS, ISM, microphone array, wideband, sub-band magnitude-squared coherence, wind noise, narrowband MUSIC

## Abstract

Parameters estimation of sequential movement events of vehicles is facing the challenges of noise interferences and the demands of portable implementation. In this paper, we propose a robust direction-of-arrival (DOA) estimation method for the sequential movement events of vehicles based on a small Micro-Electro-Mechanical System (MEMS) microphone array system. Inspired by the incoherent signal-subspace method (ISM), the method that is proposed in this work employs multiple sub-bands, which are selected from the wideband signals with high magnitude-squared coherence to track moving vehicles in the presence of wind noise. The field test results demonstrate that the proposed method has a better performance in emulating the DOA of a moving vehicle even in the case of severe wind interference than the narrowband multiple signal classification (MUSIC) method, the sub-band DOA estimation method, and the classical two-sided correlation transformation (TCT) method.

## 1. Introduction

Intelligent transportation and unmanned systems have made new demands to the parameter estimation of sequential movement events of vehicles, such as noise insensitive, scalable, and portable implementation, etc. Fortunately, microphone arrays bring new potential solutions to deal with these problems. Microphone arrays are widely used in civilian and military fields, such as noise reduction and signal enhancement [1,2,3,4], hearing aid [5,6], outdoor wind noise measurement [7,8], acoustic source localization and tracking [9,10,11,12], weapon classification and shooter localization [13,14], and so on. In the last few years, due to the development of the micro-electro-mechanical system (MEMS) technology, small aperture arrays have shown their potential superiority in terms of portability and easy deployment, especially in the field. Therefore, small aperture arrays have received more and more attention, both in industry and academia.

Recently, a small aperture MEMS microphone array system has been introduced in our earlier work [15] for direction finding for the sequential movement events of vehicles. Moreover, we have conducted a series of researches about small aperture microphone arrays and obtained some positive results, for example, acoustic target intrusion detection method [16,17], acoustic target classification method [18], and acoustic source localization method [19,20]. However, the robustness of these methods needs to be further improved in the field, especially under the influence of wind noise. To get a good bearing tracking performance, a robust direction-of-arrival (DOA) estimation method is crucial. In this paper, we will propose a wind noise robust DOA estimation method based on a small aperture microphone array system.

The mechanism and power spectrum of outdoor wind noise have been deeply studied in many works [21,22,23,24,25]. Morgan and Raspet have concluded, “The dominant source of wind noise in outdoor microphones is the pressure fluctuations that are caused by the velocity fluctuations of the incoming flow”. References [21] and [22] have indicated that wind induced noise focuses on low frequency, especially below 1 kHz. Moreover, the acoustic detection and tracking of ground vehicles also use the signal of low frequency band. Therefore, it is a challenging problem to design a wind noise robust DOA estimation method for the sequential movement events of vehicles.

From [23], wind noise changes the characteristics of the low-frequency bands of the detected acoustic signal quickly and temporally. It is difficult to separate the target acoustic signal and the wind noise from a single channel acoustic signal, both in the time domain and the frequency domain. Many researches show that the wind noise is nearly incoherent [8,25]. Fortunately, we can distinguish them by computing the magnitude squared coherence (MSC) of two selected microphones.

Independent from apertures, the MSC of the wind noise (0~4 kHz) is close to zero, while the MSC of the corresponding target acoustic signal is close to 1 [26]. Due to this difference, the spatial coherence is used to select the frequency, which is less affected by the wind noise and to estimate the DOA of a moving vehicle in [19]. Moreover, the MSC is extended from wideband to sub-band, and meanwhile the sub-band magnitude squared coherence (SMSC) DOA estimation method is designed for the vehicle bearing tracking. However, we found that it is not enough to estimate DOA robustly only by using the sub-band with maximum SMSC value alone.

In this paper, we propose a multiple high SMSC sub-bands weighting strategy and integrate it into the moving vehicle DOA estimation method of our previous work [20]. As we know, most of the DOA estimation algorithms can be divided into two classes: Maximum Likelihood (ML) based and covariance matrix decomposition based. In recent years, most of the DOA estimation research works are concentrated on ML based algorithms such as [27,28,29,30]. Although the ML based methods can get good estimation accuracy when the signals are correlated, it needs a huge computation and long run time, which is not proper for a real-time embedded platform using in the wild environment. For another class of DOA estimation methods, we have compared their advantages and disadvantages in our previous work [19]. As a result, the MUSIC method is selected to estimate the DOA after sub-bands are weighted in our developed microphone array system.

To verify the proposed method, we have carried out field experiments to check out the actually achievable performance. The results show that the proposed method has better robustness to the uncorrelated wind noise than the narrow-band MUSIC method, sub-band method [20] and classical wideband TCT method [31].

The remainder of this paper is organized as follows: Section 2 describes sequential data modeling for moving vehicles bearing tracking. Section 3 proposes the SMSC and briefly introduces the famous MUSIC method and the incoherent signal-subspace method, which would be used in the fourth section and the fifth section. Section 4 mainly analyzes the SMSC characteristics of the acoustic signals of the moving vehicles and the wind noise. Section 5 describes our proposed weighted sub-band DOA estimation method. Section 6 first introduces our designed small aperture MEMS microphone array system and then shows the field experiment results to verify the good performance of our proposed method. Finally, Section 7 concludes this paper.

## 2. Sequential Data Modeling for Bearing Tracking

**Figure 1:**
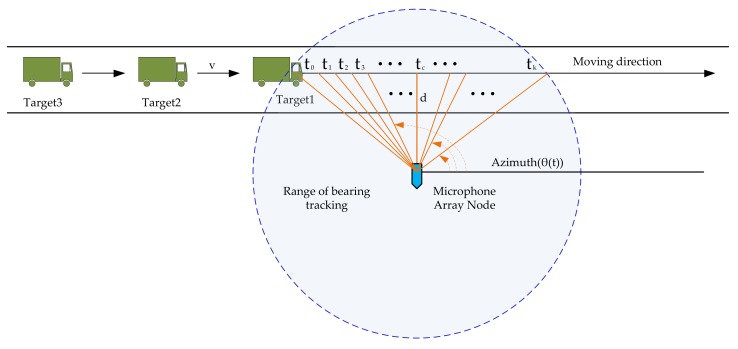
The observation scene of sequential data modeling for bearing tracking.

**Figure 2:**
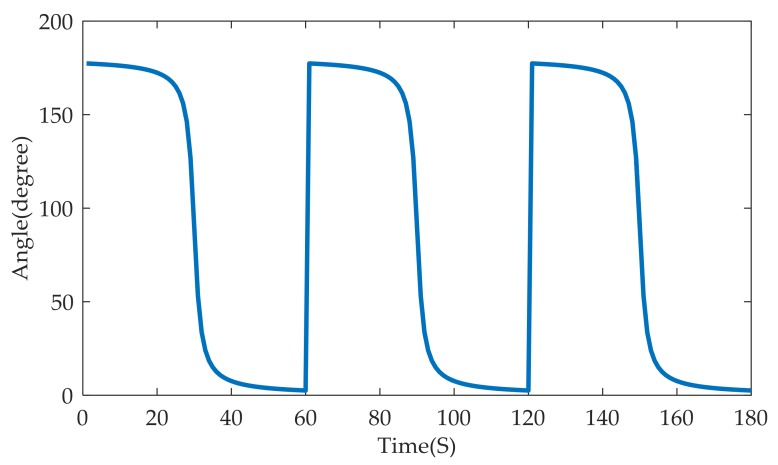
An ideal direction-of-arrival (DOA) curve of three vehicles successively passing a microphone array node.

Bearing tracking of sequential movement events of vehicles by a microphone array can be approximated as a sequential data modeling. Figure 1 shows the data observation scene for multiple targets passing a Microphone Array (MA) node. Assuming that the velocity of a vehicle (denoted as *v*) is uniform in the sampling instant time, where *t_c_* represents the moment that a target is passing the closest point of approach (CPA) and *d* represents the distance between the MA node and the lane center. Therefore, the DOA of a target passing the MA node can be described as Equation (1).

(1) $$Azimuth(\theta (t))=\pi /2+\arctan(v(t_{c}-t)/d),t=t_{0},t_{1},\cdots ,t_{k},$$

where *t*_0_, *t*_1_, …, *t_k_* represents multiple discrete observation time in the whole process of bearing tracking, and θ(t) is the observed azimuth at time *t*. Note that θ(t) is relative to the horizontal direction from left to right. Without any interference, an ideal DOA curve of three vehicles successively passing a MA node from left to right is shown in Figure 2.

As can be seen from Figure 2, the DOA ranges from 0 to 180° in this situation. When a target is far at the left side, the DOA is nearly 180° and it is gradually decreased to 0 when the target is travelling to the right side of the MA node. The ideal DOA curve is very smooth when there is no interference. However, in the real world, there always exists some interference, including instrument noise, wind disturbance, which greatly declines the DOA estimation precision. Therefore, it is especially important to improve the robustness of the DOA estimation method in the wild environment.

## 3. Subspace Based Localization Method

### 3.1. Sub-Band Magnitude-Squared Coherence (SMSC)

We first list some notations that will be commonly used in this paper.

- The matrices are indicated by uppercase bold letters and vectors by lowercase bold letters.
- The superscript * denotes the conjugate of a complex number.
- The superscript *H* denotes the conjugate transpose of a matrix.
- The superscript *T* denotes the transpose of a matrix.
- The italic *E*[·] denotes the statistical expectation.
- *f* denotes the frequency of Hertz and *ω* is the corresponding radial frequency, *ω* = 2*πf*.

The magnitude-squared coherence (MSC) between two signals *x*(*t*) and *y*(*t*) is defined as [26]:

(2) $$\gamma_{xy}(f)=\frac{p_{xy}{\left(f\right)}^{*}p_{xy}(f)}{p_{xx}(f)p_{yy}(f)},$$

where *p*_xy_(*f*) is the cross-power spectral density of *x*(*t*) and *y*(*t*). *p*_xx_(*f*) and *p_yy_*(*f*) are the auto-power spectral density, respectively. *γ_xy_*(*f*) is the MSC value at frequency *f* for *x*(*t*) and *y*(*t*). The MSC is a frequently used signal processing technique that returns real values that are between 0 and 1 to indicate how relative between two time domain signals *x*(*t*) and *y*(*t*). If *x* and *y* are completely uncorrelated, *γ_xy_*(*f*) = 0; if *x* is strictly correlative with *y*, then *γ_xy_*(*f*) = 1.

The sub-band MSC (SMSC) is used to evaluate the sub-band correlation. Suppose the wideband frequency of *x*(*t*) and *y*(*t*) can be divided into *J* uniformly distributed sub-bands, and then the *i*th sub-band MSC is defined as Equation (3) [20].

(3) $$\gamma_{xy}(f_{i})=\frac{p_{xy}{\left(f_{i}\right)}^{*}p_{xy}(f_{i})}{p_{xx}(f_{i})p_{yy}(f_{i})},i=1,2,\cdots ,J,$$

where *f_i_* is the central frequency of the *i*th sub-band. To calculate auto-power and cross-power spectral densities for *x*(*t*) and *y*(*t*), the Welch’s averaged modified periodogram method [32] is typically used. The time-domain signals *x*(*t*) and *y*(*t*) are first divided into *N* time windows of the same length (i.e., with the same number of samples). Then, the auto-power and cross-power spectral densities in each window can be calculated by discrete Fourier transform (DFT). The final MSC and SMSC can be obtained by averaging all of the corresponding values of MSC and SMSC in *N* time windows.

### 3.2. The MUSIC Method

The MUSIC method is a well-known and classical signal parameters estimation method. Different from those methods that directly process the covariance matrix of received array data, such as minimum variance method [33,34], maximum entropy method [35], conventional beam forming method [36], it performs eigenvalue decomposition on the covariance matrix of the observed signal and divides this matrix into signal subspace and noise subspace. By making use of the orthogonality between these two subspaces, the MUSIC can estimate the incident direction of targets.

Consider *P* narrowband signals in the field far from the array are impinging on an *M*-element array, then the received array signals can be expressed as:

(4) X=AS+N,

where *X =* [*x*_1_(*t*), *x*_2_(*t*), …, *x_M_*(*t*)]*^T^* is the measured signal of the *M*-dimensional array. *S =* [*s*_1_(*t*), *s*_2_(*t*), …, *s_p_*(*t*)]*^T^* represents the incident signals of *P* targets. *N =* [*n*_1_(*t*), *n*_2_(*t*), …, *n_M_*(*t*)]*^T^* is the additive noise. *A =* [*α*(*θ*_1_), *α*(*θ*_2_), …, *α*(*θ*_P_)] is the array manifold matrix. Each column of *A* is also referred to as steering vector and represents the array response to each incident signal. The matrix element *α_ij_* depends on the *i*th array element, its position relative to the origin of the coordinate system and its response to the *j*th incident signal. Once the array structure is determined and the time delays between array elements, the array manifold matrix is almost fixed. Suppose the noise signals are Gaussian distributed and are irrelevant to the source signals, then the correlation matrix of received array data can be expressed as:

(5) $$R=E[XX^{H}]=AE[SS^{H}]A^{H}+\sigma^{2}I$$

Due to the mutual independence of noise and signal, the above covariance matrix can be divided into signal subspace and noise subspace, respectively. By performing eigenvalue decomposition of *R*, we have

(6) $$R=U_{S}\Sigma_{S}U_{S}^{H}{+U}_{N}\Sigma_{N}U_{N}^{H},$$

where U*_S_*∑*_S_*$U_{S}^{H}$ represents the signal part and U*_N_∑_N_*$U_{N}^{H}$ represents the noise part. Concretely, the covariance matrix can also be expressed as *R* = $\overset{M}{\underset{i=1}{\sum }}\lambda_{i}u_{i}u_{i}^{H}$, where there are total *M* eigenvalues *λ*_1_, *λ*_2_, …, *λ_M_*, and the corresponding eigenvectors are u_1_, u_2_, …, u*_M_*. Sort the eigenvalues in descending order, then the eigenvectors corresponding to the *P* largest eigenvalues *λ_i_* > *σ*^2^ (*i =* 1, 2, *…*, *P*) constitute the signal subspace U*_S_* = [u_1_, u_2_, …, u*_P_*], while the eigenvectors corresponding to eigenvalues *λ_i_* ≈ *σ*^2^(*i = P +* 1, *P +* 2, *…*, *M*) constitute the noise subspace U*_N_* = [u*_P_*_+1_, u*_P_*_+2_,…,u*_M_*]. ∑*_S_* and ∑*_N_* are diagonal matrix and constructed by eigenvalues (*λ*_1_, *λ*_2_, …, *λ_P_*) and (*λ_P+_*_1_, *λ_P+_*_2_, …, *λ_M_*), respectively.

Ideally, the signal subspace composed of steering vectors is orthogonal to the noise subspace [37], which is expressed in the following equation:

(7) $$\alpha^{H}(\theta_{i})U_{N}=0,i=1,2,\cdots ,P$$

The MUSIC spectrum function is defined as:

(8) $$P_{MUSIC}=\frac{1}{\alpha^{H}(\theta )U_{N}U_{N}^{H}{}_{}\alpha (\theta )}$$

When Equation (7) is satisfied, the MUSIC spectrum *P_MUSIC_* at *θ_i_* is the largest. Theoretically, the MUSIC method is just using Equation (8) to distinguish different source incident signals by finding every peak of the MUSIC spectrum. Accordingly, each peak value relates to a DOA that can minimize the left part of Equation (7).

The above derivation is based on very ideal conditions and consumptions. In practical situations, the general method is to use some statistical techniques on the received signal. We can get many snapshots of the measured signal and use the statistical mean value to approximate the ideal value. Thus, the maximum likelihood estimation of the covariance matrix is given as:

(9) $$\hat{R}=\frac{1}{L}\sum_{l=1}^{L}X_{l}X_{l}{}^{H},$$

where *L* is the number of snapshots and *X_l_* represents the observed signal of the *M*-element array. Similarly, by eigenvalue decomposition of $\hat{R}$, we can get the estimated noise subspace ${\hat{U}}_{N}$*.* In practical situations, the steering vector *α*(*θ*) is not completely orthogonal to ${\hat{U}}_{N}$. However, we can search all of the possible *θ* to find the peak value of MUSIC spectrum. The final estimated DOA using MUSIC method can be expressed as

(10) $$\theta_{MUSIC}=\arg_{\theta }\min\alpha^{H}(\theta ){\hat{U}}_{N}{\hat{U}}_{N}^{H}\alpha (\theta )$$

### 3.3. Incoherent Signal-Subspace Method (ISM)

The incoherent signal-subspace method (ISM) [38] is first applied to wideband DOA estimation by decomposing wideband signals into many narrowband signals. Then, narrowband DOA estimation methods can be used on each decomposed narrowband signal. The results from all of the sub-bands are combined to get the final wideband DOA estimation.

Suppose that the wideband signal is split into total *J* sub-bands in frequency domain. The MUSIC method is applied on each sub-band. Then, the estimated covariance matrix of the *i*th sub-band can be given by Equation (11).

(11) $${\hat{R}}_{x}(f_{i})=\frac{1}{L}\sum_{l=1}^{L}X_{l}(f_{i})X_{l}{}^{H}(f_{i}),1\leq i\leq J$$

Similar to MUSIC, by proceeding eigenvalue decomposition on every sub-band covariance matrix, each sub-band signal is divided into signal subspace and noise space. Besides, the sub-band MUSIC spatial spectrum can also be calculated according to Equation (8). Then, we can obtain the wideband MUSIC spatial spectrum by averaging all sub-bands spatial spectrums, which can be expressed as

(12) $$P_{ISM}(\theta )=\frac{1}{\frac{1}{J}\sum_{i=1}^{J}\alpha^{H}(f_{i},\theta ){\hat{U}}_{N}{\hat{U}}_{N}^{H}\alpha (f_{i},\theta )}$$

## 4. Analysis of Vehicle Acoustic Signal and Wind Noise

The measured vehicle acoustic signals that are acquired in the field are mainly composed of the target acoustic signal and ambient wind noise. References [21,25] have pointed out that the dominate source of wind noise in outdoor microphones is the pressure fluctuations caused by the velocity fluctuations of the incoming flow. This phenomenon can seriously affect the microphone and cannot be completely removed by a fine-designed wind shelter.

In order to analyze the main characteristics of vehicle acoustic signals and turbulent wind noise, several typical sample signals are collected from Chongming Island, Nanjing and other places of China mainland. To avoid the involving of other noise sources, the selected experimental places are flat, open, and far away from the residential areas. We construct a signal acquisition system with a laptop, DAQ signal acquisition card and a MEMS microphone array. The INMP504 (InvenSence, Sunnyvale, CA, USA), whose signal-to-noise ratio can reach up to 65 dBA, is used to build the microphone array. Moreover, the acquisition card NI9239 (National Instruments, Austen, TX, USA) can simultaneously perform a 4-channel 24-bit synchronous data collection, with no more than 50 kHz sampling rate. The actual sampling rate is set to be 8192 Hz because the acoustic energy of the vehicle mainly targets focuses below the frequency 2 kHz. The microphone array node is placed 10 m away from the road center as shown in Figure 1. The wind speed is recorded by an ultrasonic anemometer, which is deployed beside the array during the experiments.

**Figure 3:**
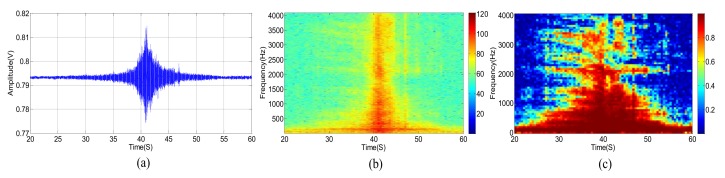
(**a**) Acoustic signal of a vehicle without wind. (**b**) Power spectrum of a vehicle without wind. (**c**) SMSC of the vehicle’s power spectrum without wind.

**Figure 4:**
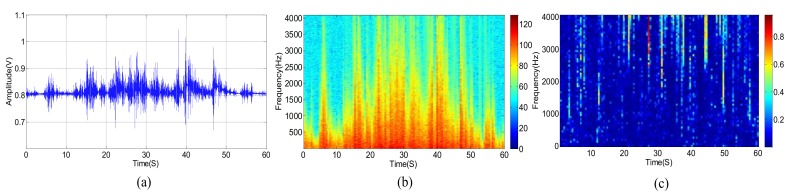
(**a**) Acoustic signal of wind noise. (**b**) Power spectrum of wind noise. (**c**) SMSC of the wind noise.

**Figure 5:**
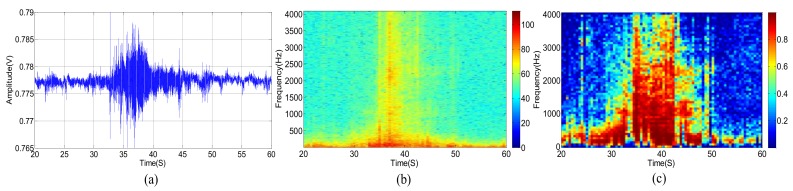
(**a**) Acoustic signal of a vehicle in the presence of wind noise. (**b**) Power Spectrum of a vehicle in the presence of wind noise. (**c**) Sub-band magnitude squared coherence (SMSC) of the vehicle’s spectrum signal in the presence of wind noise.

The original acoustic signal and its power spectrum of a passing vehicle without wind are shown in Figure 3a,b, respectively. We divide the observed wideband signal, whose frequency ranges from 0 to 4096 Hz, into equally spaced 64 sub-bands. Figure 3c shows the SMSC of each sub-band acoustic signal from two different microphones. Correspondingly, the three kinds of signals, as shown in Figure 3, are presented in Figure 4 and Figure 5, respectively, for the cases when there are only wind noise and when there are both vehicle signal and wind noise.

According to Figure 3, when the vehicle is approaching the microphone array, the signal amplitude is increasing and the spectrum range is widening gradually. The signal amplitude reaches the peak when the vehicle moves to the nearest position, and spectrum range also extends to the full band. The original signal amplitude decreases gradually while the vehicle is moving far away from the microphone array. At the same time, the spectrum range also becomes narrow slowly. When the vehicle is approaching but far away from the microphone array, there are only a few low-frequency sub-bands but with high SMSC, as depicted in Figure 3c. When the distance between the vehicle and the microphone array gets closer and closer, more and more sub-bands have higher SMSC value. Almost all sub-bands have relatively high SMSC when the vehicle arrives at the nearest point to the microphone array. When the vehicle is leaving far away from the microphone array, only low-frequency sub-bands have high SMSC again.

As shown in Figure 4, the power spectrum of wind noise in low frequency region changes rapidly at different times. However, the SMSC of wind noise is quite stable and very close to zero, especially in low frequency bands. Seeing from Figure 5, the vehicle acoustic signal is polluted by wind noise. In this case, the power spectrum is greatly damaged, especially in high frequency bands. However, the SMSC is not influenced that seriously. Although the SMSC of some sub-bands, especially the low frequency sub-bands, is nearly 0, the SMSC of most bands are still approaching to 1, which can easily be used to distinguish the source signal from wind noise.

From Figure 5, we can see that the wind noise can severely damage the original vehicle acoustic signal. When the selected frequency is greatly deteriorated, the DOA estimation result will deviate from the true value when using narrowband DOA estimation methods (such as narrowband MUSIC). Other classical methods, such as TCT, is relatively robust, the DOA estimation performance still degrades heavily when several non-continuous frequencies are damaged. The traditional sub-band method uses spatial spectrums of all the sub-bands. However, as shown in Figure 5c, some sub-bands are greatly damaged. These contaminated sub-bands can destroy the final DOA estimation result. Fortunately, there still exist some high SNR sub-bands. We can select these sub-bands with high SNR to estimate DOA which is similar to [9,39]. The main difference is that we use SMSC to evaluate the SNR. The larger the SMSC is, the higher the SNR is, and vice versa. Thus, we can search the SMSC of all the sub-bands and select a few top sub-bands with high SMSC. By weighting these sub-bands, we can output more accurate DOA estimation results. The next section will carefully present our proposed method.

## 5. The Weighted Sub-Band DOA Estimation Method

In Section 4, we analyze the spatially correlated characteristics of target acoustic signals under the wind noise. Inspired by the idea of wideband DOA method for ISM estimation, we also divide the target acoustic signal into several sub-bands in the frequency domain. The turbulent wind causes different sound pressure fluctuation on each sub-band. Therefore, the sub-band that is severely affected by the wind noise will have lower SNR and SMSC, while the sub-band is less affected by the wind noise will have rather higher SNR and SMSC. If the contaminated sub-band is used to estimate the DOA, the performance will be seriously deteriorated. In order to dismiss those low SNR sub-bands, we set a threshold TH for the SMSC. Thus, only those sub-bands that exceed the threshold are selected out. These sub-bands, which have relatively high SNR, are then weighted to calculate the spatial spectrums. The final estimated DOA of a moving vehicle can be found out by searching the spectrum peak on the selected sub-band spatial spectrums.

Suppose that there are *K* sub-bands selected for weighting from total *J* sub-bands, and then the weight of the selected *i*th sub-band *ω_i_* can be calculated by Equation (13):

(13) $$\omega_{i}=\frac{\gamma_{xy}(f_{\left(i\right)})}{\sum_{i=1}^{K}\gamma_{xy}(f_{\left(i\right)})},i=1,2,\cdots ,K\;,$$

where *γ_xy_*(*f*_(*i*)_) denotes the *i*th SMSC, which is calculated by Equation (3). When considering that the high SMSC usually has larger SNR and can give a high degree of confidence for DOA estimation, thus in our method, the sub-band with higher SMSC is assigned a larger weight. By weighting the sub-bands with high SMSC, it is inclined to get more accurate estimated DOA and to improve the robustness compared with other methods based on MUSIC.

The final MUSIC spatial spectrum using the weighted sub-bands is then calculated by Equation (14):

(14) $$P(\theta )=\frac{1}{\sum_{i=1}^{K}\omega_{i}\alpha^{H}(f_{\left(i\right)},\theta ){\hat{U}}_{N}{\hat{U}}_{N}^{H}\alpha (f_{\left(i\right)},\theta )},$$

where ***α***(*f*_(*i*)_,*θ*) denotes the steering vector of the signal subspace of the *i*th sub-band. When all of the *J* sub-bands are selected out and the received signals have the same correlation on all sub-bands (all sub-bands have the same SMSC), then *ω_i_* = 1/*J* and the above function will have the same form of the typical ISM, as expressed in Equation (12). In other words, the proposed weighted sub-band DOA estimation method is a generalized method for sub-band DOA estimation and ISM is a special form of ours.

In particular, we adopt ISM to implement weighting process on multiple sub-bands in this paper. Similarly, the weighting process also can apply to the Coherent Signal-subspace Method (CSM). Since the MEMS microphone array aperture is only 68 mm, to avoid the spurious peaks during implementation of the MUSIC method, the spectrum range is limited to no more than 2.5 kHz. When compared with the previous work [19,20], the proposed method improves the robustness of the target DOA estimation using narrowband MUSIC in the wild environment.

**Figure 6:**
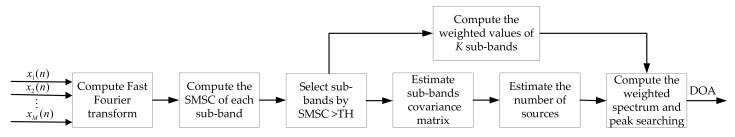
The block diagram of the weighted sub-band DOA estimation method.

The block diagram of the weighted sub-band DOA estimation method is shown in Figure 6. In accordance with Figure 6, our proposed method can be divided into the following steps:

1. Calculate the frequency domain signals for all *M* arrays using FFT on the original observed time domain signals.
2. Select two channels of microphone signals and calculate their SMSC for all of the sub-bands in the frequency domain. Sort all of the SMSC in descending order, and select the best *K* sub-bands with *K* largest SMSC. *K* is the number of sub-bands, whose SMSC is greater than the threshold TH. TH is an empirical value.
3. For the selected *K* sub-bands, calculate the weight for each sub-band.
4. Perform eigenvalue decomposition to get the noise subspace for each sub-band. Estimate the number of sources according to the sources number estimation criteria, such as MDL criterion.
5. Calculate the weighted MUSIC spatial spectrum from the *K* sub-bands and obtain the final DOA by spectrum peak searching.

## 6. Field Experiments

**Figure 7:**
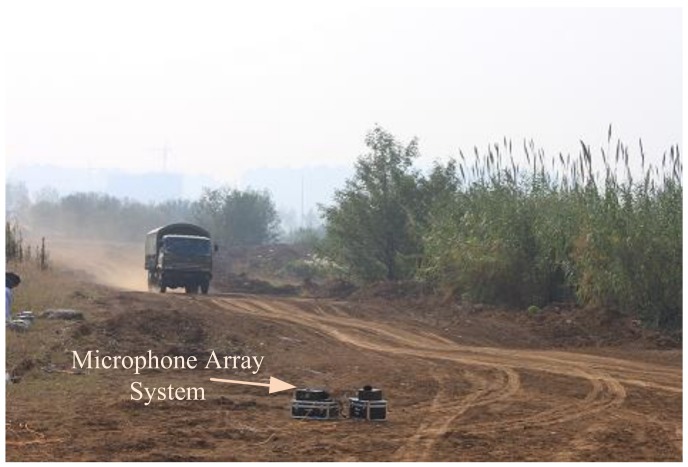
Photograph of the experimental environment.

**Figure 8:**
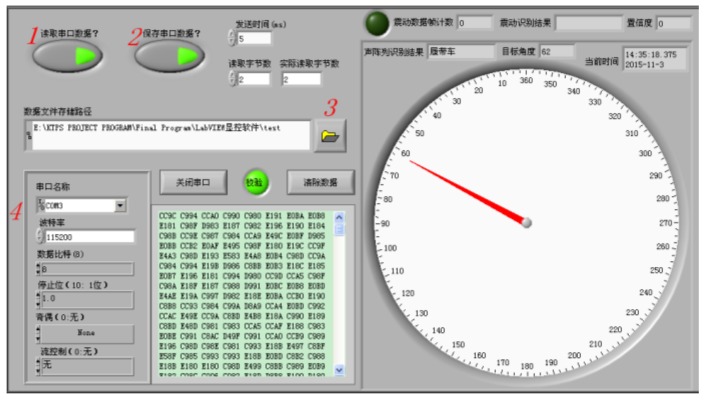
Control interface of real-time DOA and data acquisition.

In order to verify the anti-wind noise performance of the proposed method, experimental studies have been performed on Chongming Island (the third biggest islands in China) and a suburban district around Nanjing. The field test scene is shown in Figure 7 when a vehicle is passing the MEMS microphone array system. Figure 8 shows the PC (Personal Computer) user interface, which can demonstrate the real-time DOA by UART in a LabVIEW 8.5 programming environment.

First, we will introduce our hardware design for the MEMS microphone array system. Then, the experimental results using this system is described.

### 6.1. Hardware Architecture of The MEMS Microphone Array System

**Figure 9:**
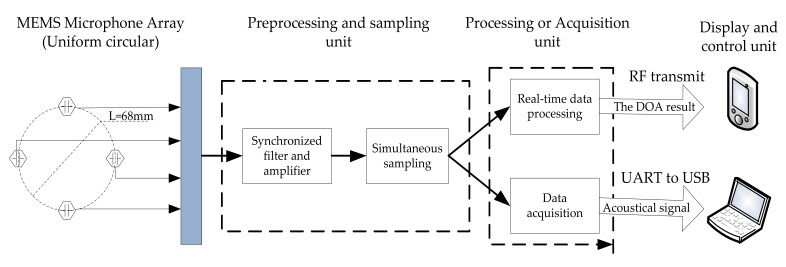
Block diagram of the MEMS Microphone Array System.

Our MEMS microphone array system consists of the following four units: MEMS microphone array, preprocessing and sampling unit, processing or acquisition unit, and display and control unit. The block diagram of the system is shown in Figure 9.

We choose a 4-element uniform circle array (UCA) with a diameter *L* = 68 mm. The MEMS microphone array converts the collected acoustic signals to electric signals and sends them to the preprocessing and sampling unit. This unit can complete various acoustic signal processing, including filtering and amplification, then through the analog digital converter (ADC) to complete the synchronous sampling conversion of multiple acoustic signals. There are two operating modes: processing or acquisition. When in mode 1, the target detection, classification, and DOA estimation is executed. When in mode 2, only four channel acoustic signals are sampled and sent out in real time. Accordingly, the display and control unit also has two operating modes. In mode 1, the target information is transmitted to a personal digital terminal device and the original data is transferred via USB cable to a portable laptop in mode 2.

**Figure 10:**
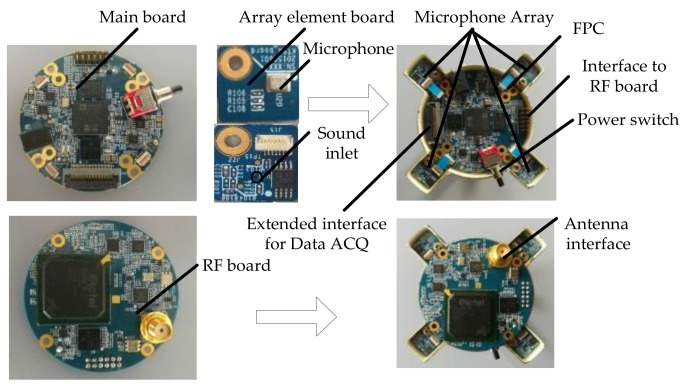
Photograph of the micro-electro-mechanical system (MEMS) microphone array system, array aperture is 68 mm.

As shown in Figure 10, our microphone array system includes a main board, four array element boards, and a radio frequency (RF) board. The main board and the array element board are connected through a Flexible Printed Circuit (FPC), and the RF board is connected with the main board through a pin connector. The main board adopts a DSP ADSP21479 (Analog Devices, Norwood, MA, USA) as the core processor, and adopts a four-channel, 16-bit ADC MAX11043 (Maxim Integrated Products, San Jose, CA, USA). Four array element boards are placed evenly on the edge of the process board. All of the array element boards have the same PCB (Printed Circuit Board) layout and routing design. This kind of design can minimize the orientation error that is caused by the interchannel inconsistency between microphone arrays. Besides, it is easy to replace any damaged MEMS microphone after a long-term working in the field. The microphone chooses INMP504 (InvenSence, Sunnyvale, CA, USA), of which the signal-to-noise ratio (SNR) can reach 65 dBA. The RF board is responsible for transmitting the detected target information in mode 1.

### 6.2. Experiments Result

**Figure 11:**
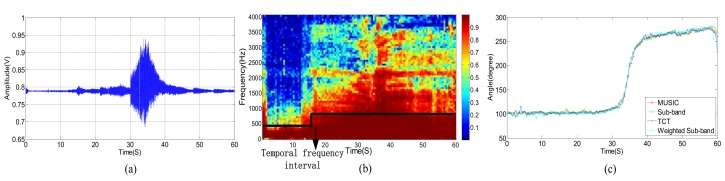
(**a**) Acoustic signal of a vehicle passing the microphone array approximate no wind. (**b**) The sub-band correlation of (**a**). (**c**) Vehicle tracking approximate no wind.

During the experiments, we compared the performance of our proposed method with narrowband MUSIC method [19], sub-band method [20] and classical TCT method. Each signal is split by sliding window, and the window length is 4096 with no overlap in 8192 Hz sampling frequency. The bandwidth of TCT, sub-band method, and weighted sub-band method are selected from (30~2500) Hz. Besides, to identify the useful sub-band of the signal, we check whether the spatial coherence is above the threshold. When considering the detecting distance of the vehicle, the threshold of weighted sub-band method is set to 0.5. Figure 11a shows the acoustic signal when the vehicle passes through the location of the microphone array system. Figure 11b shows the correlation of each sub-band in the process of vehicle, and the SMSC of the frame are approximately equal to 1, then the proposed weighted sub-band method is converted to the ISM method. Figure 11c shows the DOA estimation results of the four methods in the process of a vehicle passing under the condition that no wind disturbance. At this time, the four curves coincide perfectly.

**Figure 12:**
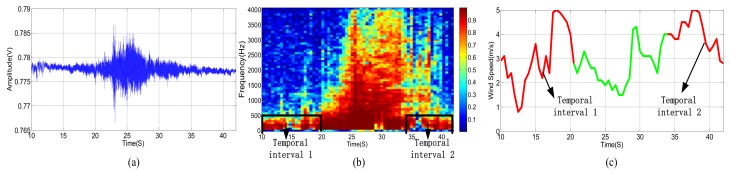
(**a**) Acoustic signal of a car passing the microphone array at level 4 wind power. (**b**) The sub-band correlation of (**a**). (**c**) Wind speed situation of (**a**).

In order to test the performance of the proposed method in the presence of wind noise, we choose a small car as the test target, which emits less sounds and is more vulnerable to wind noise than a heavy or medium duty car. Observing the two original acoustic signals that are shown in Figure 11a and Figure 12a, it is clear that the acoustic signal of the car is smaller. Figure 12b shows how the sub-band correlation of a small car changes during passing the UCA at level 4 wind power. An ultrasonic anemometer was placed near the microphone array to record the wind speed in real time. We record the moment when the target is passing the UCA and analyze the corresponding wind speed data. Figure 12c shows the variations of wind speed during the target passing the UCA.

**Figure 13:**
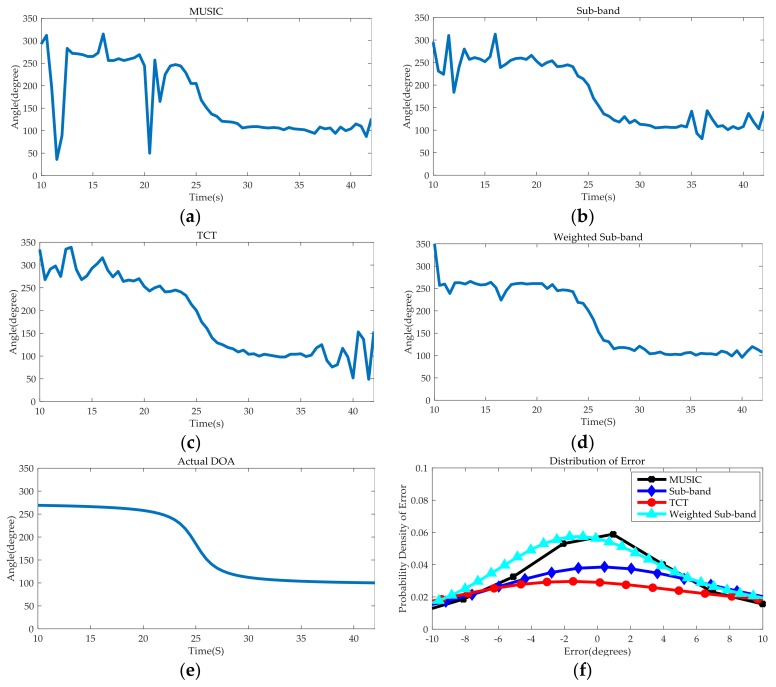
(**a**) The DOA estimation results of MUSIC method at level 4 wind. (**b**) The DOA estimation results of Sub-band method at level 4 wind. (**c**) The DOA estimation results of TCT method at level 4 wind. (**d**) The DOA estimation results of Weighted Sub-band method at level 4 wind. (**e**) The actual DOA. (**f**) The distributions of the DOA errors for four methods.

The performance to suppress wind noise of the aforesaid four DOA estimation methods is shown in Figure 13. As shown in Figure 12, the wind speed in the temporal interval 1 and the temporal interval 2 accelerates and drastically changes. As a result, the correlations between the sub-band in the low frequency parts are severely deteriorated, and the influence of each sub-band is quite different. The SMSC in this region varies randomly, from 0 to approximately 1, in comparison with Figure 11b where the target passing by, the SMSC of all the sub-bands in this temporal frequency interval are approximately equal to 1.

Since the narrowband MUSIC method [19] chooses the fixed frequency within the bandwidth to calculate manifold matrix, the DOA estimation results will deviate significantly when the area near the fixed frequency is heavily contaminated by wind noise, which can be proved by the few large deviation in the Temporal interval 1 and Temporal interval 2 in Figure 13a.The sub-band method [20] selects the sub-band that has the largest SMSC for DOA estimation. However, the problem is that when the divided sub-band is too narrow, the result will have a large deviation, and when the divided sub-band is too wide, it cannot effectively suppress the turbulent wind noise interference in the discontinuous area of the sub-band. Thus, several significant transitions occur in Temporal interval 1 and Temporal interval 2 in Figure 13b. Figure 13c shows that when the target passing the CPA point, the curve of TCT method is the smoothest among all of the curves. Although there are some fluctuations in Temporal interval 1 and Temporal interval 2, it is obviously better than the narrowband MUSIC [19] and sub-band method [20]. Figure 13d shows the entire tracking process of the designed weighted sub-band method when the target passes through the microphone array. Even in the two intervals with severe wind noise disturbance, the curve is still very smooth and the only major fluctuation of the DOA occurs around the 16th seconds. From Figure 12b, we can find that the number of sub-bands of which the threshold is bigger than 0.5 in the bandwidth range is small, the limited number of sub-bands involved in the weighting process causes this problem. Figure 13e is actual DOA change curve of our experiment. Take Figure 13e as the base results, the distributions of the DOA errors for four methods are shown in Figure 13f. The absolute mean and standard variance of the error are also given in Table 1. As shown in these results, the weighted sub-band method provide the most robust and accurate (smallest standard variance) DOA estimates.

## 7. Conclusions

In this paper, we propose a robust DOA estimation method that is designed for the sequential movement events of vehicles. We carefully analyze the correlation characteristics of the vehicle signal, respectively, with and without wind disturbance. Therefore, by selecting the sub-bands with high SMSC, we obtain the sub-bands with high SNR. Referring to the idea of ISM, we calculate the MUSIC spectrum using these sub-bands to enhance the robustness of DOA estimation. In comparison with the narrowband MUSIC method, the sub-band method, and classical TCT method, the weighted sub-band method is the best one for the DOA estimation with the presence of wind noise. By selecting the proper number of sub-bands to participate in the weighted calculation, we can effectively control the amount of computation. Therefore, the proposed method can run on a resource-limited embedded processor, which has been verified in the designed small aperture microphone array system. Field experiments have also been conducted to further test the actual achievable performance for the proposed method. The results demonstrate that our proposed method has the best robustness when compared with other three methods in terms of the absolute mean error and standard variance.

## Figures and Tables

**Table 1:** DOA Error Statistics for Various Method.

	MUSIC	Sub-Band	TCT	Weighted Sub-Band
Absolute Mean Error (deg)	18.59699	12.58701	15.21465	6.605762
Standard Variance of Error (deg)	47.651059	20.27157	22.06171	9.686766

## References

[1] Kaneda Y., Ohga J. (1986). Adaptive microphone-array system for noise reduction. IEEE Trans. Acoust. Speech Signal Process..

[2] Marro C., Mahieux Y., Simmer K.U. (1998). Analysis of noise reduction and dereverberation techniques based on microphone arrays with postfiltering. IEEE Trans. Speech Audio Process..

[3] Wang L., Cavallaro A. (2017). Microphone-array ego-noise reduction algorithms for auditory micro aerial vehicles. IEEE Sens. J..

[4] Hioka Y., Kingan M., Schmid G., Stol K.A. (2016). Speech enhancement using a microphone array mounted on an unmanned aerial vehicle. Proceedings of the 2016 IEEE International Workshop on Acoustic Signal Enhancement (IWAENC).

[5] Wang A., Yao K., Hudson R.E., Korompis D., Lorenzelli F., Soli S.F., Gao S. (1996). A high performance microphone array system for hearing aid applications. Proceedings of the 1996 IEEE International Conference on Acoustics, Speech, and Signal Processing, ICASSP-96.

[6] Zohourian M., Enzner G., Martin R. (2018). Binaural Speaker Localization Integrated Into an Adaptive Beamformer for Hearing Aids. IEEE/ACM Trans. Audio Speech Lang. Process..

[7] Bass H.E., Raspet R., Messer J.O. (1995). Experimental determination of wind speed and direction using a three microphone array. J. Acoust. Soc. Am..

[8] Wilson D.K., White M.J. (2010). Discrimination of wind noise and sound waves by their contrasting spatial and temporal properties. Acta Acust. United Acust..

[9] Pham T., Fong M. Real-time implementation of MUSIC for wideband acoustic detection and tracking. Proceedings of the SPIE AeroSense 97: Automatic Target Recognition VII.

[10] Pham T., Sadler B.M. Wideband Array Processing Algorithms for Acoustic Tracking of Ground Vehicles. http://www2.ece.ohio-state.edu/~randy/Microphone_Reading_List/pham_sadler.pdf.

[11] Jang Y., Kim J., Kim J. (2015). The development of the vehicle sound source localization system. Proceedings of the 2015 Asia-Pacific Signal and Information Processing Association Annual Summit and Conference (APSIPA).

[12] Bao Q., Luan F., Yang J. (2017). Improving the accuracy of beamforming method for moving acoustic source localization in far-field. Proceedings of the 2017 10th International Congress on Image and Signal Processing, BioMedical Engineering and Informatics (CISP-BMEI).

[13] Sallai J., Hedgecock W., Volgyesi P., Nadas A., Balogh G., Ledeczi A. (2011). Weapon classification and shooter localization using distributed multichannel acoustic sensors. J. Syst. Arch..

[14] Fernandes R.P., Apolinário J.A., Ramos A.L.L. (2017). Bearings-only aerial shooter localization using a microphone array mounted on a drone. Proceedings of the 2017 IEEE 8th Latin American Symposium on Circuits & Systems (LASCAS).

[15] Zhang X., Huang J., Song E., Liu H., Li B., Yuan X. (2014). Design of small MEMS microphone array systems for direction finding of outdoors moving vehicles. Sensors.

[16] Guo F., Huang J., Zhang X., Cheng Y., Liu H., Li B. (2015). A two-stage detection method for moving targets in the wild based on microphone array. IEEE Sens. J..

[17] Zu X., Guo F., Huang J., Zhao Q., Liu H., Li B., Yuan X. (2017). Design of an Acoustic Target Intrusion Detection System Based on Small-Aperture Microphone Array. Sensors.

[18] Huang J., Zhang X., Guo F., Zhou Q., Liu H., Li B. (2015). Design of an acoustic target classification system based on small-aperture microphone array. IEEE Trans. Instrum. Meas..

[19] Zhang X., Song E., Huang J., Liu H., Wang Y., Li B., Yuan X. (2014). Acoustic source localization via subspace based method using small aperture MEMS arrays. J. Sens..

[20] Guo F., Liu H., Huang J., Zhang X., Zu X., Li B., Yuan X. (2016). Design of a direction-of-arrival estimation method used for an automatic bearing tracking system. Sensors.

[21] Morgan S., Raspet R. (1992). Investigation of the mechanisms of low-frequency wind noise generation outdoors. J. Acoust. Soc. Am..

[22] Wuttke J. (1992). Microphones and wind. J. Audio Eng. Soc..

[23] Nelke C.M., Vary P. (2014). Measurement, analysis and simulation of wind noise signals for mobile communication devices. Proceedings of the 2014 14th International Workshop on Acoustic Signal Enhancement (IWAENC).

[24] McGuinn R.S., Lauchle G.C., Swanson D.C. (1997). Low flow-noise microphone for active noise control applications. AIAA J..

[25] Douglas Shields F. (2005). Low-frequency wind noise correlation in microphone arrays. J. Acoust. Soc. Am..

[26] Carter G., Knapp C., Nuttall A. (1973). Estimation of the magnitude-squared coherence function via overlapped fast Fourier transform processing. IEEE Trans. Audio Electroacoust..

[27] Tengtrairat N. (2017). Blind 2D signal direction for limited-sensor space using maximum likelihood estimation. Asia-Pac. J. Sci. Technol..

[28] Tengtrairat N., Woo W.L. (2017). Blind 3D sound source direction using stereo microphones based on time-delay estimation and polar-pattern histogram. Proceedings of the Information Technology (INCIT).

[29] Wang L., Cavallaro A. (2017). Time-frequency processing for sound source localization from a micro aerial vehicle. Proceedings of the 2017 IEEE International Conference on Acoustics, Speech and Signal Processing (ICASSP).

[30] Lee J.Y., Hudson R.E., Yao K. (2014). Acoustic DOA estimation: An approximate maximum likelihood approach. IEEE Syst. J..

[31] Yoon Y.S., Kaplan L.M., McClellan J.H. (2006). TOPS: New DOA estimator for wideband signals. IEEE Trans. Signal Process..

[32] Welch P. (1967). The use of fast Fourier transform for the estimation of power spectra: A method based on time averaging over short, modified periodograms. IEEE Trans. Audio Electroacoust..

[33] Capon J. (1969). High-resolution frequency-wavenumber spectrum analysis. Proc. IEEE.

[34] Hirakawa M., Tsuji H., Sano A. (2001). Computationally efficient DOA estimation based on linear prediction with Capon method. Proceedings of the 2001 IEEE International Conference on Acoustics, Speech, and Signal Processing, ICASSP’01.

[35] Ables J.G. (1974). Maximum entropy spectral analysis. Astron. Astrophys. Suppl. Ser..

[36] Malioutov D., Cetin M., Willsky A.S. (2005). A sparse signal reconstruction perspective for source localization with sensor arrays. IEEE Trans. Signal Process..

[37] Schmidt R. (1986). Multiple emitter location and signal parameter estimation. IEEE Trans. Antennas Propag..

[38] Wax M., Shan T.J., Kailath T. (1984). Spatio-temporal spectral analysis by eigenstructure methods. IEEE Trans. Acoust. Speech Signal Process..

[39] Pham T., Sadler B.M. (1996). Adaptive wideband aeroacoustic array processing. Proceedings of the 8th IEEE Signal Processing Workshop on Statistical Signal and Array Processing, Cat. No. 96TB10004.

